# Equal-Volume Strength Training With Different Training Frequencies Induces Similar Muscle Hypertrophy and Strength Improvement in Trained Participants

**DOI:** 10.3389/fphys.2021.789403

**Published:** 2022-01-05

**Authors:** Håvard Hamarsland, Hermann Moen, Ole Johannes Skaar, Preben Wahlstrøm Jorang, Håvard Saeterøy Rødahl, Bent R. Rønnestad

**Affiliations:** Section for Health and Exercise Physiology, Inland Norway University of Applied Sciences, Lillehammer, Norway

**Keywords:** skeletal muscle, resistance training, training frequency, hypertrophy, strength, trained individuals

## Abstract

The main goal of the current study was to compare the effects of volume-equated training frequency on gains in muscle mass and strength. In addition, we aimed to investigate whether the effect of training frequency was affected by the complexity, concerning the degrees of freedom, of an exercise. Participants were randomized to a moderate training frequency group (two weekly sessions) or high training frequency group (four weekly sessions). Twenty-one participants (male: 11, female: 10, age: 25.9 ± 4.0) completed the 9-week whole-body progressive heavy resistance training intervention with moderate (*n* = 13) or high (*n* = 8) training frequency. Whole-body and regional changes in lean mass were measured using dual-energy x-ray absorptiometry, while the vastus lateralis thickness was measured by ultrasound. Changes in muscle strength were measured as one repetition maximum for squat, hack squat, bench press, and chest press. No differences between groups were observed for any of the measures of muscle growth or muscle strength. Muscle strength increased to a greater extent in hack squat and chest press than squat and bench press for both moderate (50 and 21% vs. 19 and 14%, respectively) and high-frequency groups (63 and 31% vs. 19 and 16%, respectively), with no differences between groups. These results suggest that training frequency is less decisive when weekly training volume is equated. Further, familiarity with an exercise seems to be of greater importance for strength adaptations than the complexity of the exercise.

## Introduction

Resistance training is an essential tool in the pursuit of athletic performance (McGuigan et al., 2012) and for improving health (El-Kotob et al., 2020). To optimize the effects of resistance training, the manipulation of several factors, primarily training volume, load, and frequency, is central (American College of Sports Medicine, 2009). The current recommendations on training frequency, defined as the number of sets or training sessions on a given muscle group performed within a given timeframe, have been criticized for being based on limited evidence (Grgic et al., 2018; Ralston et al., 2018; Schoenfeld et al., 2019). The latest meta-analyses suggest a limited role for training frequency, given that the weekly training volume is kept identical between groups (Grgic et al., 2018; Ralston et al., 2018; Schoenfeld et al., 2019). However, in the current literature, studies isolating frequency by keeping total weekly volume matched between groups are limited; most include untrained participants and compare training frequencies of one to three sessions per week. As trained individuals are more likely to have greater training frequencies than less trained individuals, and the frequencies used are likely to be greater than those investigated in most studies (Strömbäck et al., 2018), a need for more research on this group has been expressed (Grgic et al., 2018; Ralston et al., 2018). To our knowledge, there are ten published studies on volume equated resistance training frequency in trained individuals published to date (McLester et al., 2000; Schoenfeld et al., 2015; Brigatto et al., 2018; Colquhoun et al., 2018; Gentil et al., 2018; Gomes et al., 2019; Lasevicius et al., 2019; Saric et al., 2019; Zaroni et al., 2019; Johnsen and van den Tillaar, 2021). Five of these studies have focused on higher frequencies than 3 days per week (Colquhoun et al., 2018; Gomes et al., 2019; Saric et al., 2019; Zaroni et al., 2019; Johnsen and van den Tillaar, 2021). Zaroni et al. (2019) reported greater muscle hypertrophy but no differences in strength gain when comparing one (lower body) and two (upper body) weekly sessions with five sessions per week in young, trained men. Apart from this study, there seems to be no difference in muscular adaptations when comparing one and five (Gomes et al., 2019), two and four (Johnsen and van den Tillaar, 2021), or three and six (Colquhoun et al., 2018; Saric et al., 2019) weekly training sessions in young trained men.

Although the current evidence for a beneficial effect of higher training frequencies in trained individuals is weak, it is based on small studies with limited power to detect small but meaningful differences between protocols. Thus, there is a need for more data to be able to make precise and evidence-based recommendations. In addition, there are several theoretical advantages to increased training frequency. The protein synthetic response to a training stimulus is considered to last for at least 24–48 h after a bout of resistance exercise in untrained individuals (MacDougall et al., 1995; Phillips et al., 1997; Burd et al., 2011). A higher training frequency may therefore allow for more time with a net positive protein balance and greater muscular adaptations to resistance training. In contrast to untrained individuals, exercise-induced elevations of protein synthesis appear to last only about 24 h after resistance exercise in resistance-trained individuals (Damas et al., 2015). Thus, the advantages of higher training frequencies may increase with training status. Further, it has been suggested that distributing training volume across several days may reduce fatigue during the sessions (Dankel et al., 2016) and reduce recovery time between sessions (Pareja-Blanco et al., 2020). This may allow for greater training loads, potentially resulting in superior muscular adaptations to resistance training. Lastly, more frequent neuromuscular stimuli may optimize motor learning, increasing strength through neurological factors (Shea:2000dt). This may be more pronounced in complex multi-joint, free weight lifts, with greater degrees of freedom, compared with simpler single joint or machine-based exercises. Although neurological adaptations are primarily expected to occur at the beginning of training, antagonist inhibition has been shown to improve also after years of exercise (Balshaw et al., 2019). To the best of our knowledge, no study has yet explored whether exercise complexity influences the potential benefits of training frequency in trained individuals.

Therefore, the current study aimed to compare the effects of two and four weekly volume equated heavy resistance training sessions on gains in muscle mass and -strength in resistance-trained men and women. Further, we investigated whether the effect of training frequency was influenced by the complexity of the exercises. We hypothesized that a volume equated weekly training frequency of four sessions per week would result in greater muscular adaptations and strength gains than two sessions per week. We further hypothesized that the benefits of a higher training frequency would be greater in more complex exercises (squat and bench press) compared to less complex exercises (hack squat and chest press).

## Materials and Methods

### Ethical Approval

The study was performed according to the ethical standards established by the *Declaration o*f Helsinki 2013 and was approved by the Local Ethical Committee at the Inland Norway University of Applied Sciences (20/03749) and pre-registered in a Norwegian public database (Norwegian Center for Research, project number 300667). All participants signed an informed consent form before participation.

### Participants

Thirty-four moderately resistance-trained men and women volunteered to participate in the study. To be included in the study, participants had to be between 18 and 35 years of age, free of injury, performed one resistance-training workout per week on average over the last 6 months, and be familiar with the powerlifting exercises squat and bench press. Participants were randomized into a high-frequency group (HF) (*n* = 17) and a low-frequency group (LF) (*n* = 17) stratified by sex, age, years of resistance training experience, and 1 repetition maximum (RM) results from the first test. Participants who had more than 10 days without a workout or were not able to complete 95% of the planned sets were excluded (*n* = 3). Due to the ongoing sars-CoV-2 pandemic, the number of participants who were allowed to exercise simultaneously was controlled and training times were strict. Participants who were unable to attend these times, due to quarantine (*n* = 3) or sars-CoV-2 infection (*n* = 1), were excluded (*n* = 3). Three participants were unable to complete the study due to injuries and three participants dropped out of the study due to personal reasons. Thus, 21 participants were included in the analysis (HF: *n* = 8, LF:*n* = 13).

### Experimental Design

The intervention consisted of a 9-week training period (see Table 1). While the total amounts of weekly sets and training load (RM) were identical, the HF and LF groups performed 4 and 2 sessions per week, respectively. Thus, HF performed half the training volume of the LF per workout. The training period was divided into three blocks based on RM number: week 1–3: 12RM, week. If failure was reached before the intended repetition range, the resistance was quickly reduced to allow for the remaining repetitions to be completed. If participants were able to complete more than the intended repetitions the set was performed to failure and the resistance was increased at the next set. Each week, the exercise order was rotated to balance the number of workouts, starting with a more complex exercise (squat and bench press) and a less complex exercise (hack squat and chest press in a machine). Four minutes of rest was given between each set. To equate the warm-up sets across each week, LF performed two sets (12 reps of 30% 1RM and 12 reps of 50% 1RM), whereas HF performed four sets (2×12 reps of 30% 1RM and 2×12 reps of 50% 1RM). There was a strong focus on completing all sets in every workout. All sessions were supervised by trained personnel. To counteract a potential effect of differences in protein intake participants were provided 20 g of whey protein mixed in water after each workout. To balance the protein supplementation, the protein was also ingested the day after workouts in the low-frequency group. Before and after the training period, participants performed a set of 1RM tests (squat, hack squat, bench press, and chest press) and underwent a dual-energy x-ray absorptiometry scan (DXA) and an ultrasound scan of the vastus lateralis muscle from both legs.

**TABLE 1:** Training protocol.

Group	Day 1 exercise: sets	Day 2 exercise: sets	Day 3 exercise: sets	Day 4 exercise: sets	Total weekly sets	Repetitions and load
**High** **frequency**	Squat: 2	Hack squat: 2	Squat: 2	Hack squat: 2		First workout: 70% 1RM Week 1–3: 12RM Week 4–6: 10RM Week 7–9: 8RM
	Hack squat: 1	Squat: 1	Hack squat: 1	Squat: 1		
	Bench press: 2	Chest press: 2	Bench press: 2	Chest press: 2		
	Chest press: 1	Bench press: 1	Chest press: 1	Bench press: 1		
	Lat pulldowns: 2	Seated row: 2	Lat pulldowns: 2	Seated row: 2		
**Total workout sets**	**8**	**8**	**8**	**8**	**32**	
**Low** **frequency**	Squat: 4		Hack squat: 4			
	Hack squat: 2		Squat: 2			
	Bench press: 4		Chest press: 4			
	Chest press: 2		Bench press: 2			
	Lat pulldowns: 4		Seated row: 4			
**Total workout sets**	**16**		**16**		**32**	

### Testing Procedures

The participants were instructed to avoid exercise and strenuous physical activity for 48 h before all tests. Instructional videos explaining the 1RM testing procedures were emailed to and watched by all participants before testing. Before the 1RM test, participants warmed up for 5 min on a rowing ergometer (Concept 2 inc., Vermont, VT, United States) with an intensity of 9–11 on the 6–20 Borg-scale (Borg, 1982), followed by two sets of 20 walking lunges and two sets of 10 shoulder rotations using a wooden dowel. On the first 1RM test, individual settings were established for each participant, and identical settings were used on subsequent testing. The 1RM tests were performed by completing single repetitions with increasing load and 4 min of rest between each repetition. The goal was to reach 1RM on the tenth repetition. After attaining 1RM for squat and bench press, participants had a 30-min break with a small meal before completing the 1RM tests for hack squat and chest press. The meal was identical at all 1RM tests within an individual participant but differed between participants. The squat and bench press were performed in a Tteka BN-02 combo (TTEKA company, Montreal, QC, Canada). The execution of the squat and bench press was performed according to the standardization of the International Powerlifting Federation (2021) technical rules, except the grip width in the bench press, which was set to be 81 cm from the fifth finger to the fifth finger. The hack squat was performed in a Cybex International Hack Squat (Cybex International, Inc., Massachusetts, MA, United States). Joint angles were measured using a goniometer. The correct depth was set as a hip joint angle of **≤** 90° and a knee joint angle smaller than 90°. Foot placement was adjusted to 22.5, 27.5 (middle of the plate), or 32.5 cm to achieve the intended hip and knee angles. The correct depth for each participant was noted on a vertical measuring band attached to the hack squat. Chest press was performed in a Cybex Converging Plate Loaded Chest Press (Cybex International, Inc., Massachusetts, MA, United States). The chest press started with arms in the extended position and was finished with arms returned to the extended position. A band was attached between the handles, and an accepted 1RM required the band to touch the chest at *proc. xiphoideus*. During the lift, the whole foot remained in contact with the floor, the buttocks in contact with the seat and the back, shoulder blades, and head in contact with the back support. A test leader controlled all 1RM attempts.

Body composition was determined using a dual-energy x-ray absorptiometry (DXA) (Lunar Prodigy, GE Healthcare, Madison, WI, United States) after an overnight fast and was analyzed following the protocol of the manufacturer, using the software of the manufacturer. The participants were placed in the supine position with a neutral neck position. Hands were semi-pronated and arms were placed close to the border of the measurement area to simplify the post-scan measurements. The foot position was standardized in a neutral position using a foam rubber cast (10 cm between heels) with negligible absorbance. Regions of interest were used to analyze different body regions. Dividing lines between arms and thorax were drawn through the glenohumeral joint. Dividing lines between the thorax and legs were drawn midway through the femoral neck at a 90-degree angle to the femoral neck. All DXA scans were performed by a researcher blinded to subject randomization. The intraclass correlation (ICC) for lean body mass in our lab is 0.99. The muscle thickness of m. vastus lateralis of both legs were measured using B-mode ultrasonography (SmartUs EXT-1 M, Telemed, Vilnius, Lithuania) with a 39 mm 12 MHz, linear array probe. Image depth was set to 8 cm, frequency to 12 Hz, the dynamic range was set to 72 dB, and the gain was set to 43%. Longitudinal images were obtained ∼50% distally from the trochanter major toward the femoral lateral epicondyle. Three images were captured before and after the training intervention. The probe’s position was marked on the skin and subsequently marked on a soft transparent plastic sheet superimposed on the thigh. Landmarks such as moles and scars were also marked on the plastic sheet for relocation of the probe during post-training measurements. Furthermore, pre-test images were used to locate anatomical landmarks on the post-test images to ensure the same measuring location. During analysis, pre and post-images from the same participant were analyzed consecutively using the Fiji software macro tool “Simple Muscle Architecture Analysis” (Seynnes and Cronin, 2020). The average muscle thickness of the three images from each leg was averaged, and the average of both legs at the given time point was used for further analyses. All ultrasound measures were performed by a researcher blinded to subject randomization. ICC for ultrasound measures of vastus lateralis in our lab is 0.96. After the training period, the DXA and ultrasound were measured between 48 and 96 h after the last training session.

### Statistical Analysis

Statistical analyses were performed using jamovi (version 1.6.23.0 for mac; the jamovi project, retrieved from www.jamovi.org) and GraphPad Prism (version 9.2.0 for mac; GraphPad Software, La Jolla, CA, United States). The effect of HF *vs*. LF on study variables was analyzed using ANCOVA, with post-intervention outcomes as dependent variables and baseline values and sex as covariates. Pearson’s correlation coefficients (*r*) were calculated for changes in 1RM and lean mass. The effect of HF *vs*. LF in simple and complex exercises and training volume and load were analyzed by a two-way ANOVA with repeated measures. Furthermore, the effect size (ES) was calculated as Cohen’s *d* using the mean pre-post change in HF minus the mean pre-post change in LF, divided by the pooled pre-test standard deviation (Morris, 2008). Outcomes are reported with standard deviation unless otherwise specified. A two-tailed *P*-value less than 0.05 was considered significant.

## Results

### Muscle Strength

**FIGURE 1:**
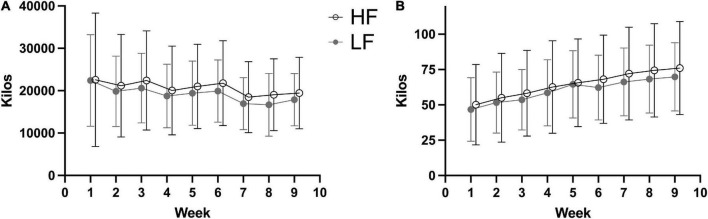
Weekly training volume **(A)** and average load **(B)** with 2 (LF) and 4 (HF) weekly sessions during a 9-week resistance training intervention.

**FIGURE 2:**
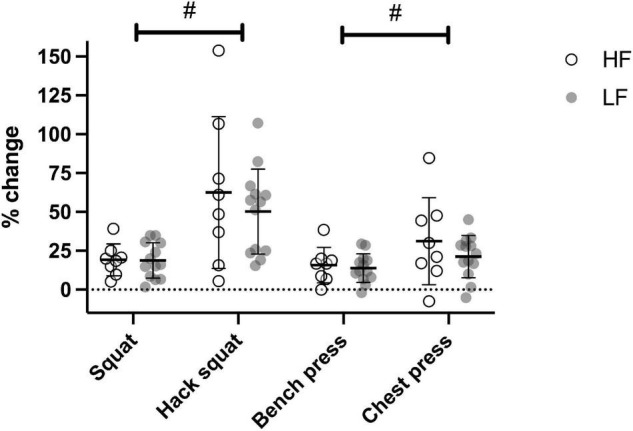
Percent changes in one repetition maximum for squat, hack squat, bench press, and chest press. Horizontal lines are averages with error bars representing SD. ^#^Difference between simple and complex exercises, *p* < 0.05.

The weekly training volume and load are displayed in Figure 1. Both groups improved 1RM in squat (HF: 16.3 ± 5.2 kg, LF: 14.8 ± 4.2 kg), hack squat (HF: 33.4 ± 13.9 kg, LF: 34.4 ± 9.7), bench press (HF: 7.5 ± 3.5, LF: 7.7 ± 3.0 kg), and chest press (HF: 15.9 ± 13.8 kg, LF: 15.0 ± 5.0 kg) during the training intervention. The improvements in 1RM did not differ between groups (see Table 2 and Figure 2).

**TABLE 2:** Comparisons between groups are based on estimated marginal means.

	ANCOVA HF vs. LF			
	Mean effect	*P*	ES	95% CI
Lean mass total	−0.1 kg	0.897	−0.07	−1.09 to 0.97
Lean mass legs	−0.4 kg	0.296	−0.54	−1.61to 0.54
Lean mass trunk	0.0 kg	0.969	−0.02	−0.99 to 1.04
Lean mass arms	0.2 kg	0.210	0.61	−0.41 to 1.63
VL thickness	−0.00 cm	0.950	−0.03	−1.01 to 0.95
1RM total	5.5 kg	0.637	0.3	−0.79 to 1.25
1RM squat	4.2 kg	0.192	0.69	−0.41 to 1.79
1RM hack squat	−1.27 kg	0.824	−0.11	−1.11 to 0.89
1RM bench press	0.7 kg	0.676	0.20	−0.80 to 1.21
1RM chest press	2.7 kg	0.554	0.26	−0.74 to 1.26

### Lean Mass and Muscle Thickness

Lean mass (HF: 1.14 ± 2.0 kg, LF: 1.46 ± 1.38 kg), lean leg mass (HF:0.35 ± 0.79 kg, LF:0.64 ± 0.63 kg), lean trunk mass (HF:0.41 ± 1.17 kg, LF:0.66 ± 0.93 kg), lean arm mass (HF:0.38 ± 0.40 kg, LF:0.18 ± 0.24 kg), and vastus lateralis thickness (HF:0.51 ± 0.22 cm, LF:0.48 ± 0.18) improved in both groups. There were no differences between groups for any of the measures of muscle growth (see Table 2 and Figure 1).

### Complex vs. Simpler Exercises

The hack squat 1RM (HF: 62.5 ± 48.8%, LF: 50.2 ± 23.6%) increased more in relative terms than the squat 1RM (HF: 19.1 ± 10.3%, LF: 18.7 ± 8.6%) in both groups (*P* < 0.01 for both). Chest press 1RM increased more in relative terms than bench press 1RM in HF (31.2 ± 27.9% and 15.8 ± 11.5%, respectively, *P* = 0.011) but not in LF (13.8 ± 7.4% and 21.3 ± 7.8%, respectively, *P* = 0.171). Combining both groups 1RM in the less complex exercises increased more than more complex exercises (Lower body: 18.9 ± 10.7% vs. 54.9 ± 36.3%, *P* < 0.00; Upper body: 14.6 ± 9.9% vs. 25.0 ± 20.2%, *P* = 0.001). The differences between squat and hack squat, and bench press and chest press did not differ between groups. There was a significant correlation between changes in 1RM for all exercises (squat – hack squat: *r* = 0.64, squat – bench press: *r* = 0.83, squat – chest press: *r* = 0.69, hack squat – bench press: *r* = 0.74, hack squat – chest press: *r* = 0.73, bench press – chest press: *r* = 0.78, *p* > 0.001 for all). There were no significant correlations between changes in muscle strength and changes in muscle mass.

## Discussion

The current study found that in moderately resistance-trained individuals distributing weekly resistance training volume into two or four workouts did not result in different outcomes for 1RM, lean mass, or vastus lateralis thickness. Further, muscle strength in complex exercises does not benefit more from a higher training frequency than in simpler exercises.

In line with most previous studies, weekly training frequency seems to be subordinate to training volume in terms of increasing muscle mass, given training volume is kept identical within each week (Colquhoun et al., 2018; Gomes et al., 2019). Our finding goes against the theoretical benefit of multiple upregulations of stimulating protein synthesis and thus increased anabolic stimulus with an increased frequency. This could result from a suboptimal training stimulus when the training volume was distributed across 4 days. However, previous studies suggest that relatively small training volumes can produce a robust muscle protein synthetic response in resistance-trained and active individuals (Burd et al., 2010, 2011). Several recent contributions may help shed more light on this discrepancy. Evidence suggests that volume-sensitive long-term adaptations of translational capacity, rather than repeated acute changes in translational efficiency, are associated with hypertrophy (Figueiredo, 2019; Hammarström et al., 2020). This is further supported by the lack of measurable differences in myofibrillar protein synthesis over 7 days with a matched training volume distributed across one or five workouts (Shad et al., 2021). Alternatively, the manipulation of resistance training variables may be secondary to other intrinsic factors. At least when a sufficient stimulus is provided, as suggested by Damas et al. (2019) who elegantly displayed remarkable stability within individuals despite manipulating resistance training variables, in contrast to a substantial between-individual variability.

It has been suggested that an increased training frequency may enhance neural adaptations to a greater extent in more complex exercises compared with simple exercises (Shea et al., 2000). Given a more complex movement pattern, we expected strength in squat and bench press to increase more compared with hack squat and chest press in the HF group when compared with the LF group. To the best of our knowledge, this is the first study to investigate this effect from a resistance training perspective. Contrary to our hypothesis, 1RM increased more in the less complex exercises. The difference was surprisingly large given the exercises had similar movement patterns and were trained with the same weekly volume. This is possibly explained by the participants having considerably more experience with squat and bench press than hack squat and chest press (see Table 3). Thus, our data suggest familiarity with an exercise to be of greater importance than the complexity of the exercise when it comes to improvements in muscle strength.

**TABLE 3:** Descriptive data of participants before and after 9 weeks of heavy resistance exercise.

Characteristic	HF pre	HF post	LF pre	LF post
N (♂/♀)	3/5	3/5	8/5	8/5
Age (years)	26.8 ± 3.9		25.5 ± 4.3	
Body mass (kg)	80.7 ± 15.7		74.6 ± 12.8	
Lean mass (kg)	53.7 ± 12.4	54.8 ± 12.4	53.7 ± 9.4	55.2 ± 12.1
Lean mass legs (kg)	18.5 ± 4.7	18.9 ± 4.4	18.2 ± 3.1	18.8 ± 4.4
Lean mass trunk (kg)	25.9 ± 5.5	26.3 ± 5.5	25.9 ± 4.8	26.6 ± 4.7
Lean mass arms (kg)	6.1 ± 2.2	6.4 ± 2.2	6.4 ± 1.6	6.6 ± 1.7
Body fat (%)	31.9 ± 4.9	31.6 ± 4.3	25.1 ± 6.9	24.4 ± 6.0
Vastus lateralis thickness (cm)	2.4 ± 0.5	2.9 ± 0.4	2.6 ± 0.4	3.1 ± 0.4
Squat 1RM (kg)	106 ± 56	122 ± 56	96 ± 37	110 ± 34
Hack squat 1RM (kg)	88 ± 61	122 ± 57	89 ± 52	123 ± 53
Bench press 1RM (kg)	70 ± 42	78 ± 40	70 ± 31	78 ± 30
Chest press 1RM (kg)	93 ± 67	108 ± 60	97 ± 52	112 ± 49
Weekly sessions squat	1.5 ± 0.9		1.3 ± 0.8	
Weekly sessions hack squat	0.3 ± 0.9		0 ± 0	
Weekly sessions bench press	1.2 ± 1.1		1.2 ± 0.9	
Weekly sessions chest press	0.5 ± 0.9		0.6 ± 0.6	
Resistance training age (years)	3.6 ± 2.2		3.5 ± 1.3	

*Weekly sessions are an estimate of the weekly average number of sessions including the exercise over the last 6 months. Values are average ± SD.*

Although the relative changes in 1RM differed between similar exercises, we observed moderate to strong correlations between these changes in all measured exercises. This may suggest that changes in e.g., both squat and hack squat 1RM to a large extent, should represent changes in leg strength as a phenomenon. However, as these correlations exist between all measured exercises, they may well be driven by the training state of the participants. It is expected that the weaker participants will improve more for all exercises during the training intervention. Accordingly, the baseline 1RM performance did correlate negatively with changes in 1RM during the intervention (from −0.83 to −0.94, *p* < 0.001 for all measures). Strength is often considered a relatively universal trait. However, the present and previous data (Harris et al., 2007) suggest that a significant part of improvements in strength are specific to the given exercise and may differ significantly between similar exercises trained identically over a training period.

The current study has several strengths, among these a full supervision of all workouts, a 100% attendance, and a direct measure of muscle growth. However, it shares the limitation of a relatively short intervention period with the rest of the current training frequency literature in trained individuals. Even with a 3-week cutback, due to corona restrictions, the current 9-week study is longer than the previous 6- to 8-week interventions (Colquhoun et al., 2018; Gomes et al., 2019; Zaroni et al., 2019; Johnsen and van den Tillaar, 2021). Training protocols with equated training volume, but different training frequencies are not expected to result in large differences in adaptations. Consequently, longer interventions may be needed to discern differences between them. Measures of muscle thickness were only obtained at one point along the vastus lateralis. Previous studies have shown non-uniform hypertrophy in the quadriceps in response to resistance exercise (Narici et al., 1996). Recently, drop sets were reported to induce greater gains in hypertrophy for the rectus femoris, but not vastus lateralis, compared with traditional resistance exercise training (Varović et al., 2021). Consequently, we cannot exclude the possibility of regional differences in hypertrophy within or between muscles. Still, any potential regional differences were not large enough to be observed with the DXA measures. Given the large intraindividual variability and limited resources, a unilateral approach to further investigate the effects of training frequency seems sensible, at least for muscle growth where cross-learning is unlikely to occur (MacInnis et al., 2017). Furthermore, if two exercise regimens are compared in a contralateral manner in resistance-trained individuals, it is our belief that potential cross-learning effects on strength too will be minimal.

## Conclusion

In conclusion, the current study did not show an effect of resistance training frequency on changes in muscle strength and muscle growth when weekly resistance training volume was kept identical in moderately resistance-trained individuals. Further, higher resistance training frequency did not result in greater improvements in strength for complex exercises compared to simpler exercises.

## Data Availability Statement

The raw data supporting the conclusions of this article will be made available by the authors, without undue reservation.

## Ethics Statement

The studies involving human participants were reviewed and approved by the Local Ethical Committee at Inland Norway University of Applied Sciences (20/03749). The patients/participants provided their written informed consent to participate in this study.

## Author Contributions

HH, HM, OS, PJ, HR, and BR contributed to the conception and design of the study. HM, OS, PJ, and HR conducted the training intervention. HH, HM, OS, PJ, and HR performed the testing. HH wrote the first draft of the manuscript. HM, OS, PJ, HR, and BR wrote sections of the manuscript. All authors contributed to manuscript revision, read, and approved the submitted version.

## Conflict of Interest

The authors declare that the research was conducted in the absence of any commercial or financial relationships that could be construed as a potential conflict of interest.

## Publisher’s Note

All claims expressed in this article are solely those of the authors and do not necessarily represent those of their affiliated organizations, or those of the publisher, the editors and the reviewers. Any product that may be evaluated in this article, or claim that may be made by its manufacturer, is not guaranteed or endorsed by the publisher.
